# Time-Resolved Spatial Distributions of Individual Components of Electroactive Films during Potentiodynamic Electrodeposition

**DOI:** 10.1021/acsphyschemau.4c00055

**Published:** 2024-09-03

**Authors:** Rachel M. Sapstead, Robert M. Dalgliesh, Virginia C. Ferreira, Charlotte Beebee, Erik Watkins, A. Robert Hillman, Karl S. Ryder, Emma L. Smith, Nina-Juliane Steinke

**Affiliations:** † Centre for Sustainable Materials Processing, Department of Chemistry, University of Leicester, Leicester LE1 7RH, U.K.; ‡ STFC ISIS Neutron and Muon Source, Rutherford Appleton Laboratory, Didcot OX11 0QX, U.K.; § CQB, Centro de Química Estrutural, Institute of Moledular Sciences, Faculdade de Ciências, Universidade de Lisboa, Campo Grande, 1749-016 Lisboa, Portugal; ∥ Neutron Scattering Division, Oak Ridge National Laboratory, Oak Ridge, Tennessee 37830, United States; ⊥ School of Science and Technology, Nottingham Trent University, Clifton Campus, Nottingham NG11 8NS, U.K.; # Institut Laue-Langevin, 38000 Grenoble, France

**Keywords:** neutron reflectivity, event mode, electrodeposition, polypyrrole, copper, interfacial structure

## Abstract

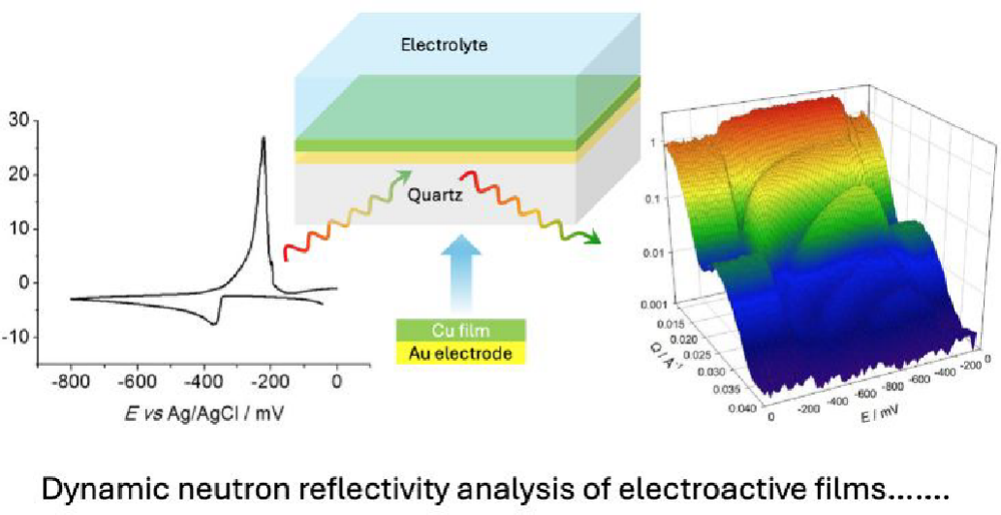

Of the attributes that determine the performance of electroactive film-based devices, the least well quantified and understood is the spatial distribution of the component species. This is critical since it dictates the transport rates of *all* the mobile species (electrons, counterions, solvent, analyte, and reactant) and the film mechanical properties (as exploited in actuator devices). One of the few techniques able to provide individual species population profiles *in situ* is specular neutron reflectivity (NR). Historically, this information is obtained at the cost of poor time resolution (hours). Here we show how NR measurements with *event mode* data acquisition enable both spatial *and* temporal resolution; the latter can be selected postexperiment and varied during the transient. We profile individual species at “buried” interfaces under dynamic electrochemical conditions during polypyrrole electrodeposition and Cu deposition/dissolution. In the case of polypyrrole, the film is homogeneous throughout growth; there is no evidence of dendrite formation followed by solvent (water) displacement. Correlation of NR-derived film thickness and coulometric assay allows calculation of the solvent volume fraction, ϕ_S_ = 0.48. In the case of Cu in a deep eutectic solvent, the complexing nature of the medium results in time-dependent metal speciation: mechanistically, dissolution does not simply follow the deposition pathway in reverse.

A combination of the opacity of common solvents to IR radiation, the negligible population of interfacial (cf. bulk solution) species, and the outcome of a 3D (laterally and vertically) spatially integrated response limits the efficacy of spectroscopic techniques for “wet” interfacial characterization. The highly penetrating nature of neutrons and the surface specificity of reflectivity measurements mean that neutron reflectivity (NR) is an ideal technique for spatial profiling of “buried” electroactive films.^1^ This capability is illustrated by *static* (5 min to 5 h) application to Li insertion into battery materials,^2^ hydrogen uptake by metal coatings used for nuclear waste containment,^3^ and film solvation phenomena accompanying anion uptake into PoT,^4^ a crown-ether functionalized conducting polymer,^5^ PVF,^6^ and PVF–PNIPAM-based (co)polymers.^7^ These highlight the ability of NR spatially to track electroinactive, nonabsorbing, interfacial minority species via isotopic substitution (“contrast variation”).

Full integration of NR into the portfolio of techniques that support electrochemistry demands *dynamic* capability. This was originally demonstrated for an (electro)chemically stable material by multiple redox cycling and coaddition of reflectivity profiles.^8^ Alternatively, this can be achieved at the cost of a restricted range of momentum transfer (Q = [4π/λ]sin θ), as demonstrated for metal electrodeposition,^9^ Li-mediated N_2_ reduction,^10^ and anion extraction from solution.^11^ The latter strategy brings data fitting challenges, recently addressed by novel data fitting algorithms.^12^ The challenge is illustrated by acquisition of long and short time scale NR profiles associated with anion uptake by PVF-based films,^11^ but only fitting of the former.

The potential for time-resolved NR to provide novel insights into interfacial phenomena has prompted exposition of instrumental design and attributes for a neutron reflectometer with kinetic capability.^13^ Combination with novel data fitting protocols^12^ motivates acquisition of time-resolved NR evidence to rationalize compositional and structural influences on the dynamics of electrochemically driven interfacial processes. We demonstrate this capability here for two strategic material scenarios: electropolymerization of a conducting polymer film and electrodeposition/dissolution of a metal.

Combination of coulometric assays of surface populations with external topography from imaging techniques^14^ and “vertically” averaged film composition from spectroscopic^15^ and acoustic^16^ techniques provides useful insights. However, these methods cannot probe (in)homogeneity in the film interior, where the majority of functionality lies. Spatial distributions of film components can be probed using reflectivity methods. Optical reflectivity (ellipsometry^15b^) can be used to estimate total film solvation but interpretation can be complicated by model selection. *In situ* X-ray reflectivity^17^ measurements are impeded by the presence of light element components (polymer/solvent) in electrochemical systems and by limited penetration depth. Conversely, neutrons are highly penetrating and “contrast variation” can be exploited through H/D substitution of solvent or film components.

The advance we describe is use of *event mode* NR data acquisition in short time scale “single shot” experiments, exemplified by film deposition. We exploit two crucial features made possible by the assignment of an individual time stamp to each detected neutron subsequent to surface reflection. First, the time resolution is selected postmeasurement, facilitating optimization of resolution vs signal:noise. Second, the time resolution can be varied within a given transient, allowing pursuit of detailed mechanistic interrogation at times (or potentials) when the system undergoes rapid change and maximization of signal:noise at times (or potentials) of slow change.

NR profiles were collected *in situ* during electrochemical film deposition. The working electrodes (area = 28 cm^2^) were ∼35 nm thick Au layers, sputter coated onto polished single crystal quartz blocks.^8,9,18^ Polypyrrole (PPy) was deposited potentiodynamically (1 mV s^–1^; 20 cycles; 36 μA cm^–2^ anodic current cap) from 0.1 M NaClO_4_/D_2_O/0.1 M h-Py. Copper deposition/dissolution was performed potentiodynamically (−0.8 < E/V < 0.0; 0.02 mV s^–1^) from 10 mM CuCl_2_ in the deep eutectic solvent Ethaline (choline chloride:ethylene glycol = 1:2). The potentiostat was an Ivium CompactStat.

NR measurements were performed on the OFFSPEC reflectometer at the ISIS Facility of the Rutherford Appleton Laboratory (Harwell, Oxford, UK). Data were acquired in event mode and “time sliced” for optimal averaging postmeasurement. NR data acquired over sequential (PPy growth) or single (Cu) cycles are presented as a function of momentum transfer, Q/Å^–1^ = (4π/λ)sin θ, in each time/potential window. The neutron wavelength range 1.5 < λ/Å < 16 and incident angle θ = 0.5° correspond to a momentum transfer range 0.007 < Q/Å^–1^ < 0.07. The collimation slits were set to give a beam footprint on the sample of 60 mm × 30 mm.

Figure 1 shows cyclic voltammograms during PPy growth (panel a) and the corresponding reflectivity profiles, R(Q), as a function of the number of growth cycles (panel b). R(Q) responses were sliced such that NR data in the last 300 mV of each cycle and in the first 300 mV of the subsequent cycle (representing reduced PPy, under conditions of no change) were combined to yield a precise film assay after each cycle. For the data subsets of the first and last cycles, we used only the first 300 mV and last 300 mV of the cycle, respectively.

**Figure 1:**
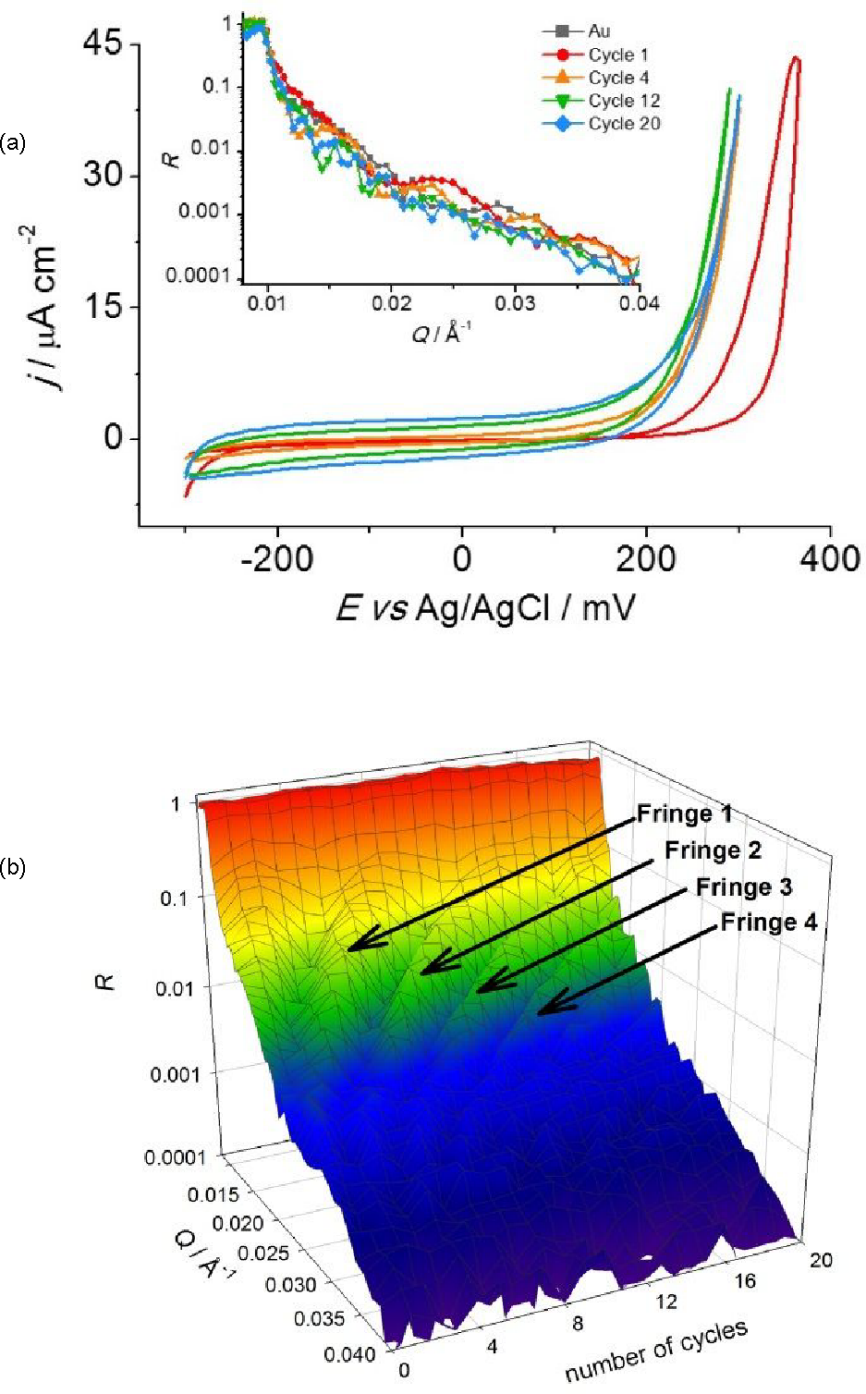
Polypyrrole potentiodynamic growth from 0.1 M NaClO_4_/0.1 M h-Py aqueous solution: (a) cyclic voltammogram at 1 mV s^–1^ (20 cycles) with I_max._ = 0.7 mA (25 μA cm^–2^) at the anodic limit, (insert: NR profiles); (b) dynamic NR profiles as functions of Q and number of potentiodynamic growth cycles. In panel (a), the potential range of 650 mV is traversed in 650 s at a scan rate of 1 mV s^–1^. In panel (b), the full potential excursion of 1300 mV means that each potentiodynamic growth cycle represents a time interval of 1300s.

The shape of a neutron reflectivity profile, R(Q), modeled as a series of layers that cause constructive interference, yields information about film structure. At low Q, total external reflection is observed, due to the substrate (quartz) through which the neutrons are transmitted having a lower scattering length density than the material from which they are reflected (D_2_O). This produces a critical edge, at Q_c_ = (16πΔNb)^1/2^, where ΔNb corresponds to the difference in scattering length densities between the two bulk phases (∼6.30 × 10^–6^ and 4.18 × 10^–6^ Å^–2^ for D_2_O and quartz, respectively). Inserting ΔNb = 2.12 × 10^–6^ Å^–2^ into the expression above, Q_c_ ∼ 0.01 Å^–1^, consistent with experiment (Figure 1b). The periodicity of the Bragg fringes is dictated by the thickness of the layers (Au electrode and polymer) and their amplitude by the scattering length density contrast and sharpness of the interfaces between layers. Noting that Q (/Å^–1^) is in reciprocal space, the progressive increase in fringe frequency (Figure 1b) qualitatively demonstrates increased film thickness after each potential cycle.

These outcomes are quantified in terms of film thickness, d = 2π/ΔQ, where ΔQ is the fringe periodicity (from Figure 1b). Figure 2 shows the resulting plot of d as a function of the growth charge, q_g_. For the extended range of film thickness explored, no single fringe was visible across the entire measurement, so a composite plot was assembled from ΔQ values for the first four fringes that appeared (and, in two cases, then disappeared) as the film grew. Linear correlation of film thickness (d) with monomer consumption (q_g_) reveals a uniform growth mechanism; this rules out the alternative scenario of rapid dendritic growth of a thick film with a low polymer segment density that fills in at longer times. The film thickness data in Figure 2 correspond to the h-PPy layer plus the gold electrode. Fits of predeposition NR data yield a gold electrode thickness of 355 Å, consistent with the intercept in Figure 2. The final thickness of the polymer layer was 2158 Å.

**Figure 2:**
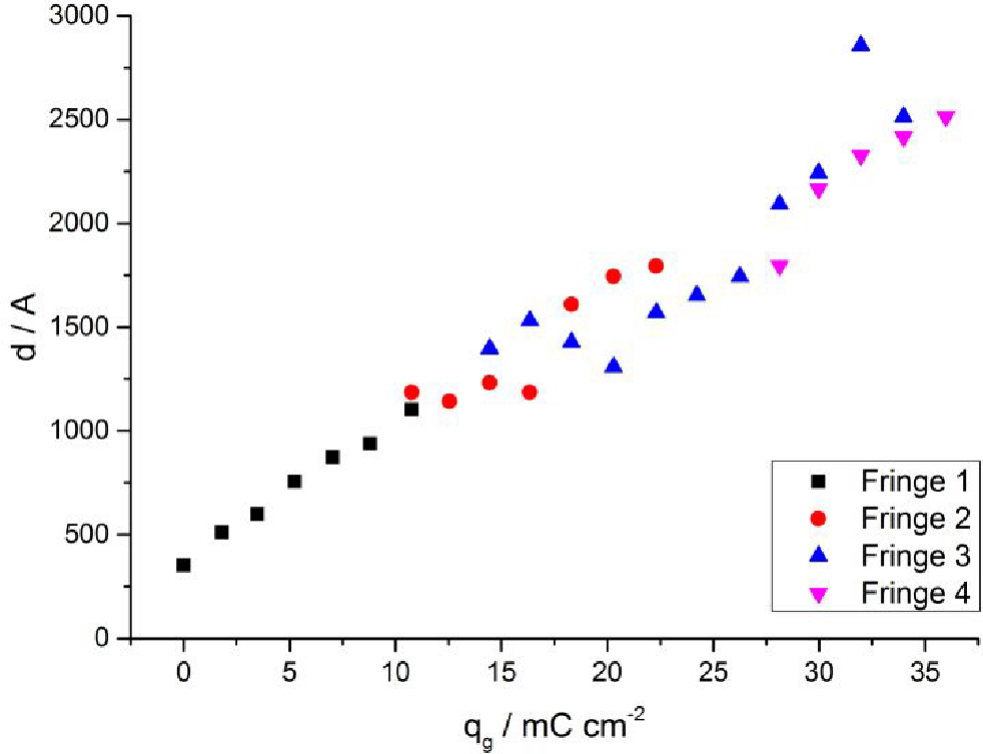
Film thickness, d, calculated from ΔQ in NR profiles obtained during the potentiodynamic growth of h-PPy as a function of growth charge, q_g_.

Coulometric assay of the spatially integrated surface population of polymer is given by Γ/mol m^–2^ = q/nFA; q/C is the charge, n is the number of electrons transferred per monomer during polymerization and consequent “doping” (for a “doping level” of 0.3, n = 2.3), A is the electrode area, and F is Faraday’s constant (96,485 C mol^–1^). For the data shown, the final polymer coverage (in terms of monomer units) was 1.62 × 10^–3^ mol m^–2^.

The observed molar volume can be calculated by combining the NR and electrochemical data via V_m(obs)_ = d/Γ. We find V_m(obs)_ = 1.33 × 10^–4^ m^3^ mol^–1^. The theoretical molar volume for a dry, compact PPy film, V_m(theor)_, is 6.9 × 10^–5^ m^3^ mol^–1^. The distinction between these values represents solvent swelling of the film: we calculate a solvent volume fraction, ϕ_s_ = 0.48. More sophisticated questions relating to temporal variation of ϕ_s_ during and after film oxidation will be explored in the future.

Figure 3 shows the cyclic voltammogram of the deposition and dissolution of Cu from CuCl_2_ in Ethaline (panel a) and corresponding reflectivity profiles, R(Q), as a function of potential, E (panel b). The NR data were cut into 20 mV potential slices (1000 s time intervals), except in the region of film dissolution, where exploiting the facility of event mode data acquisition, 5 mV potential slices (250 s time intervals) were used to enhance temporal resolution.

**Figure 3:**
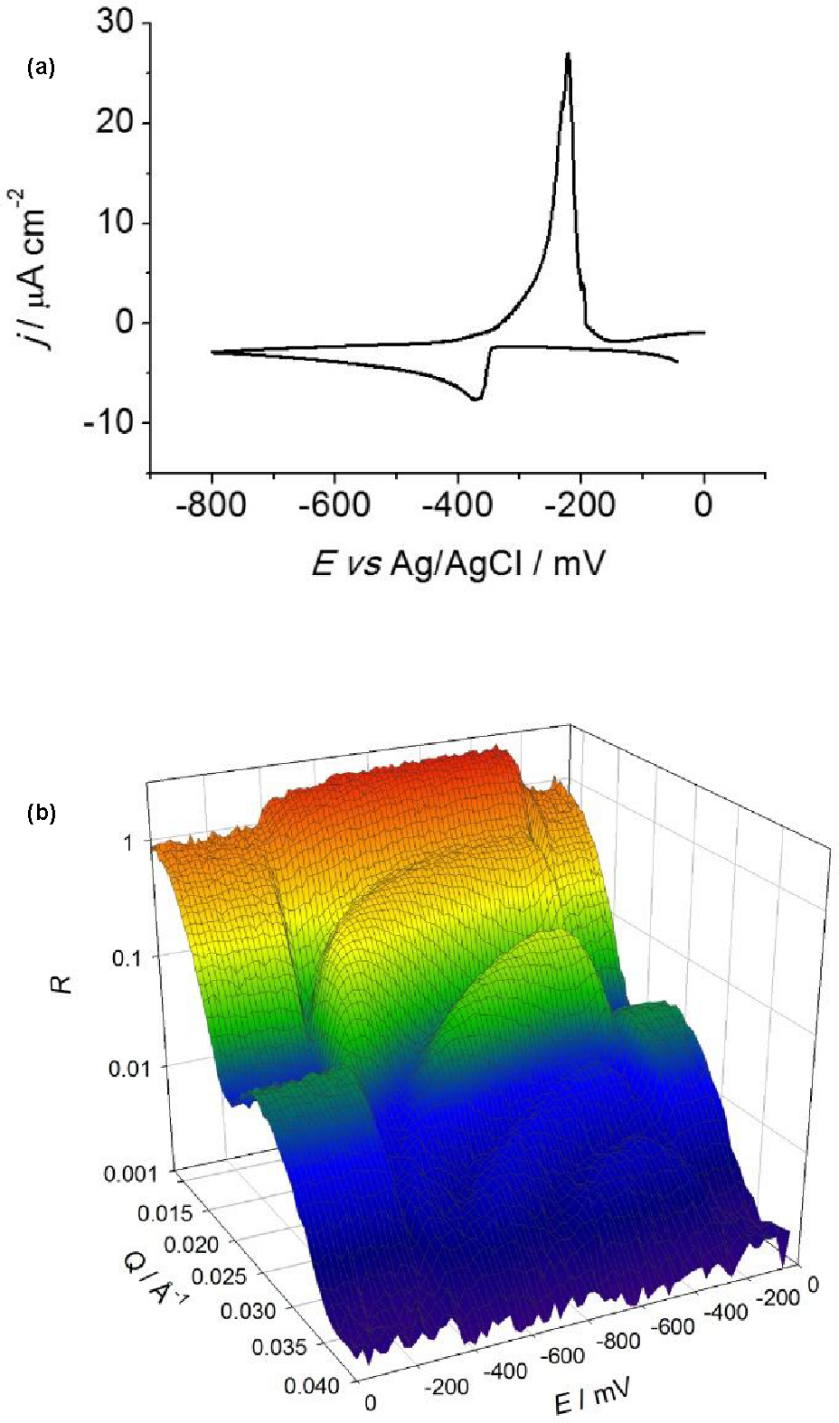
Copper potentiodynamic deposition/dissolution from 10 mM CuCl_2_ solution in Ethaline: (a) cyclic voltammogram at 0.02 mV s^–1^ between 0 and −800 mV and (b) simultaneous dynamic NR profiles as functions of Q and E.

We found four distinct regions in the R(Q) vs E plot. Between 0 and −360 mV three fringes are present, attributable to the bare Au electrode. From −360 to −800 mV in the cathodic (forward) scan and −800 to–280 mV in the anodic (reverse) scan, the frequency of the fringes increased as Cu was deposited. The amplitude of the fringes increased, as Cu has a higher scattering length density than Au (6.58 × 10^–6^ cf. 4.49 × 10^–6^ Å^–2^, respectively). Between −280 and −180 mV in the anodic scan, the fringes broadened and decreased in both frequency and amplitude as Cu was stripped from the surface. Between −180 and 0 mV in the anodic scan, the initial R(Q) profile was restored.

Figure 4 shows that, unlike the conducting polymer case, Cu deposition in this medium has two separate stages of linear growth. The first stage of deposition (0 < E/mV < −790) generated a film 450 Å thick (based on the ΔQ calculation discussed above). The d vs q plot for this first stage had a slope smaller than that of the second stage. Qualitatively we attribute this to potential-dependent involvement of two processes at the more positive end of the voltammetric sweep, Cu(II) → Cu(I) and Cu(I) → Cu(0), of which only the latter generates a film. Quantitative interpretation is ongoing, and will consider possible contributions of underpotential deposition (UPD) and precipitation of a Cu(I) species. During the second stage, involving a charge passage of 0.079 C cm^–2^, the film thickness increases by ca. 980 Å. Combining these two, on the basis of Cu(I) reduction as the sole process, we calculate an observed molar volume of V_m(obs)_ of 11 × 10^–6^ m^3^ mol^–1^. Comparison with the theoretical molar volume for a solid film of pure Cu (V_m(theor)_ = 7.09 × 10^–6^ m^3^ mol^–1^), we deduce that the film contains pores, to the extent of a solvent volume fraction of ϕ_s_ ≈ 0.34.

**Figure 4:**
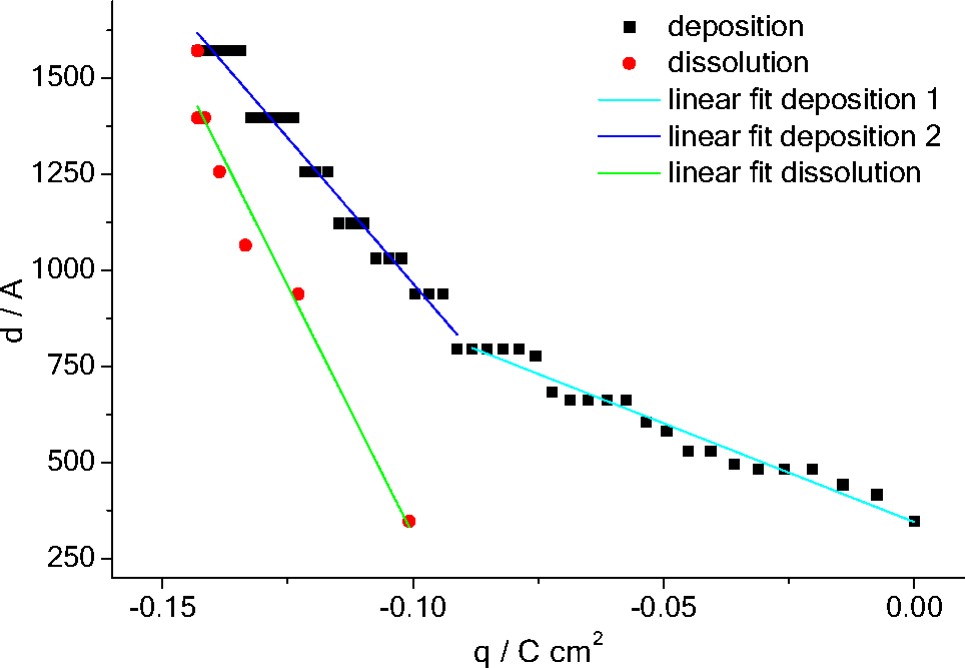
Film thickness, d, calculated from ΔQ in NR profiles obtained during the potentiodynamic deposition and dissolution of Cu as a function of charge, q.

The dissolution d vs q slope was very steep compared to that for deposition and the total charge passed during dissolution (0.042 C cm^–2^) was less than half the deposition charge. This indicates that dissolution was a one electron redox process, plausible in the high chloride medium used. The data also suggest that electrolytic dissolution of Cu at the Au interface results in mechanical detachment of some Cu(0), without charge passage.

In conclusion, the power of event mode NR data acquisition in two different dynamic electrochemical systems has been demonstrated. During potentiodynamic growth of PPy from aqueous medium, the film retains constant solvent content (ϕ_S_ = 0.48). Potentiodynamic deposition/dissolution of copper in the deep eutectic solvent Ethaline involves both Cu(I) and Cu(II) at different points, and dissolution involves some non-Faradaic loss of Cu. We suggest that event mode NR will allow real time analysis of changes in structure, composition (solvent and doping levels), and internal film dynamics on time scales commensurate with *operando* device application involving a wide range of electroactive film materials.
